# Reduced caregiving quality measured during the strange situation procedure increases child’s autonomic nervous system stress response

**DOI:** 10.1186/s13034-019-0302-3

**Published:** 2019-10-31

**Authors:** Franziska Köhler-Dauner, Eva Roder, Sabrina Krause, Anna Buchheim, Harald Gündel, Jörg M. Fegert, Ute Ziegenhain, Christiane Waller

**Affiliations:** 1 Department of Child and Adolescent Psychiatry/Psychotherapy, University Hospital of Ulm Medical University of Ulm, Steinhövelstraße 5, 89075 Ulm, Germany; 2 Department of Psychosomatic Medicine and Psychotherapy, University Hospital of Ulm, Ulm, Germany; 3 Institute of Psychology, University Innsbruck, Innsbruck, Austria; 4 Department of Psychosomatic Medicine and Psychotherapy, Paracelsus Medical University, Nuremberg General Hospital, Nuremberg, Germany

**Keywords:** Caregiving quality, Parasympathetic nervous system, Sympathetic nervous system, Strange situation procedure, Mother–child dyad

## Abstract

**Background:**

Dysfunctional maternal behavior has been shown to lead to disturbances in infant’s regulatory capacities and alterations in vagal reactivity. We aim to investigate the autonomic nervous system (ANS) response of the child during the strange situation procedure (SSP) in relation to the quality of maternal behavior.

**Methods:**

Twelve month after birth, 163 mother–child-dyads were investigated during the SSP. Heart rate (HR) and both, the parasympathetic branch (PNS) via the respiratory sinus arrhythmia (RSA) and the sympathetic branch (SNS) via the left ventricular ejection time (LVET) of the ANS were continuously determined during the SSP using electrocardiogram (ECG) and impedance cardiogram (ICG) measures. Maternal behavior was assessed by using the AMBIANCE measure.

**Results:**

The ANS response in infants of mothers with disruptive behavior compared to infants of non-disruptive mothers was significantly altered during the SSP: HR increased especially when infants of disruptive mothers were alone with the stranger (F (1, 161) = 4.15, p = .04) with a significant vagal withdrawal when being in contact with the stranger despite of presence of the mother (F (1, 161) = 5.11, p = .03) and a significant increase in vagal tone during final reunion (F (1, 161) = 3.76, p = .05). HR increase was mainly based on a decrease in LVET (F (1, 161) = 4.08, p = .05) with a maximum infant’s HR when the stranger came into the room instead of the mother.

**Conclusion:**

Both, SNS and PNS branches of the child are significantly altered in terms of an ANS imbalance, especially during contract to a stranger, in relation to dysfunctional maternal behavior. Our findings suggest the importance of supporting high quality caregiving that enables the infant to adapt adequately to stressful interpersonal situations which is likely to promote later health.

## Introduction

Early infants’ development of behavioral and physiological regulation depends mainly on the experience in social interaction with their caregivers [1–3]. Especially in stressful situations, infants’ regulatory processes are mainly influenced by the experienced interactions between infant and their caregivers [4] with mother and father acting as an external regulator of infant arousal. Caregivers are attuned to and act to soothe distress during a period when their infant has not yet developed a sufficient repertoire of regulatory capacities [5]. Especially sensitive and responsive caregiving, defined by an accurate interpretation and prompt response to infant needs, can protect infants from inordinate stress and support them by developing effective stress regulation strategies [6]. A number of empirical studies have found maternal interaction quality to be associated with individual differences in infants’ regulatory strategies [7–9]. During the 1st years of infant’s life, infants need to manage the challenging transition from external regulation of affect and internal arousal to rising levels of psychobiological regulation [10]. The concept of parental sensitivity is grounded in attachment theory: “perception of the child’s signals”, “appropriate interpretation of the signals” and “appropriate and prompt response to child’s signals” [11]. Caregiver sensitivity is critical for reducing young infants’ distress in situation of emotional arousal and may influence infants’ negative emotions in the way that infants are able to develop and practice the ability to modulate arousal by regulation [12, 13]. On the other hand, less sensitive and supportive parenting (e.g. like frightening and anxious interaction behavior) might constrain or reduce the ability of physiological and behavioral regulation development [14, 15]. In attachment theory as well as developmental theory it is suggested that the relationship between infant and parent is an important factor for the development of child’s regulatory strategies [10, 16, 17]. Numerous findings showed that the quality of parental interacting behavior especially in the 1st years of infant’s life is an essential predictor for infants’ behavioral and/or physiological regulation outcomes [18–21]. Even if it is well evaluated that maternal behavior may compensate for stressful situations, only little is known about the underlying physiological aspects that influence the child’s stress responses [6]. In recent years, bio-physiological parameters have been used to underline behavioral observations and to obtain the understanding of the interaction between behavioral and physiological systems in infancy. One of the most relevant stress-related bio-physiological measures are that of the autonomic nervous system (ANS) [22]. The ANS consists of two branches—the parasympathetic nervous system (PNS) and the sympathetic nervous system (SNS)—which demonstrates individual differences in children’s responses to emerging situations and is mainly involved in emotional as well as behavioral regulation activated by social interaction [23].

### Maternal behavior as a predictor of infant’s stress regulation related to ANS

The ANS mainly involved in emotional and behavioral reactions initiated by attachment and therefore has been widely used to investigate emotion regulation during infant development and in different psychopathologies [24–27]. Alterations in ANS are detectable far in advance of the awareness of specific emotions. However, the effects of maternal affective behavior on child’s ANS related stress reactivity have rarely been studied [28, 29]. The SNS branch is activated in response to an external threat like “fight or flight” by increasing heart rate and respiration [30]. In contrast the PNS branch has an inhibitory effect on the SNS and mediates “rest and digest” by maintaining homeostasis and regulating recovery following stress by decreasing heart rate and respiration [31, 32]. The increase in heart rate is thus influenced by both the PNS and SNS reactivity [33]. The respiratory sinus arrhythmia (RSA), determined using the interbeat intervals of the ECG and the respiratory rates derived from the ICG at a bandwidth range of 0.15–0.080 Hz [34], reflects the PNS branch of the ANS and is a common index to measure vagal functioning in young infants [35–39]. The left ventricular ejection time (LVET) is a chronotrophic SNS parameter and represents the blood ejection time of the left ventricle which is extracted from a time interval until he closure of the aortic valve in the ICG [40].

Findings on RSA stress reactivity with regard to a comparable experimental paradigm (e.g. the Still-Face Paradigm (SFP; [41]) show that lower quality of maternal behavior is associated with higher activation in infants’ RSA [42]. In detail, Moore et al. revealed associations between lower quality of maternal behavior and a decrease in infants’ RSA [9, 43–46]. Using the same paradigm in younger children shows that lower maternal sensitivity during periods of stress leads to lower PNS activation [47]. Recent findings identified RSA as suitable PNS marker compared to simple HR measures [35–39].

Several studies demonstrated increasing stability in baseline PNS during infant growth with significant associations to temperament, behavior, and health [48, 49]. PNS activity has been shown to be related preliminary to social engagement and that tonic PNS control stabilizes around 12 months of infant’s age whereas PNS stress reactivity showed high variability until older age. The mode of autonomic imbalance in response to stressful situations depends on the age of the infant as well as on its social interaction and attachment quality [27, 35, 50, 51]. In contrast to the understanding of the PNS in this context, there is limited knowledge about the SNS and its stability over time in relation to maternal attachment behavior and child’s development. The role of the SNS mediated ‘fight or flight’ response [52] in relation to attachment behavior as well as the integrated function of SNS and PNS in this context are rarely investigated [35, 53–55]. However, Oosterman and Schluengel [54, 55] used SNS measures in attachment research and emotional as well as cognitive related measurements [54, 55]. Hinnant investigated PEP in young infants and revealed significant stability over time [56] furthermore Oosterman and Schuengel [54] showed findings of differences in child’s SNS response measured by PEP from the age of 3 years [54]. Infant’s LVET during mother–child-interaction is rarely investigated. Recently, Roder et al. [57] have been identified LVET as a suitable marker to measure SNS in a 1-year-old child, since the LVET measure is frequency-related which is essential for the detection of SNS in young children.

### Maternal behavior and child’s vagal regulation

Numerous empirical studies identified a direct association between the quality of caregiving behavior and the child’s vagal regulation. For example, Moore and Calkins [45] demonstrated that infants of less sensitive and responsive mothers showed less adaptive patterns of vagal regulation resulting in higher vagal withdrawal during normal play episodes, less vagal withdrawal during stressful situations and more difficulty returning to a level of baseline vagal tone after distress [45]. Furthermore, Perry and colleagues [10] reported that maternal emotional support predicts child’s trajectory of vagal regulation. Infants of mothers with more responsiveness and sensitivity in interactive situations were found to have greater vagal withdrawal at age 3 to 4 compared to infants of mothers with lower levels of responsiveness and sensitivity [10].

Results of Calkins and colleagues revealed that negative and controlling maternal interacting behavior was also associated with a reduction of child’s vagal withdrawal [1]. In contrast to that, maternal positive touch has been shown to reduce the child’s physiological reactivity in stressful situations [58]. Further studies confirmed the association between caregiving interacting behavior and child’s vagal withdrawal. Calkins and colleagues investigated the quality of mother–child-relationships at age 2 in relation to the degree of infants’ vagal withdrawal 3 years later. They detected that with increasing quality of the mother–child-relationship child’s vagal withdrawal was significantly accentuated at later age. They found that the quality of maternal-child relationship at age 2 predicted the degree of infants’ vagal withdrawal at age 5 even after controlling for behavioral problems and vagal withdrawal at age 2, such that infants with poorer early maternal-child relationships displayed significantly less vagal withdrawal at a later age [59]. Oosterman and Schuengel [54, 55] measured infants’ autonomic reactivity in foster children and a control group and elicited less variability RSA reactivity in foster children across the episodes of the SSP [54].

Taken together, the child’s development of effective vagal regulation is predicted by the quality of maternal interacting behavior [10, 60–62].

The aim of our study was to determine the relation between child’s ANS reactivity, measured via RSA und LVET and maternal interacting quality, both based on the SSP. We hypothesize that (1) infants of mothers with disruptive behavior show higher HR during SSP, especially during episodes of maternal separation and contact with the stranger, compared to children of non-disruptive mothers. These children reveal (2) an aggravation of RSA withdrawal compared to infants of non-disruptive mothers. Concerning the SNS, we hypothesize (3) that LVET can serve as a suitable marker of SNS stress induced changes in children of disruptive mothers. Based on the LVET measures, SNS activity is hypothesized (4) to be significantly increased in children of disruptive mothers due to an increase in ANS stress response, especially in contact with the stranger, compared to the SNS activity measured in children of non-disruptive mothers.

## Material and methods

### Participants and study design

Trans-Gen is an interdisciplinary study consortium investigating the pathways leading to resilience or vulnerability in the transgenerational transmission of childhood maltreatment (CM) in a prospective approach. In a birth cohort recruited in the women’s hospital of the University Hospital of Ulm, we examined psychological, biological and social factors that positively influence the association between maternal load through CM and the infant’s cognitive and social-emotional development as well as their stress reactivity. The study was funded by the Federal Ministry of Education and Research (BMBF, 2013–2016, additional interim funding 2017) and approved by the Ethics Committee of Ulm.

Since October 2013, 533 mother–child-dyads were being recruited in the maternity unit of the women’s hospital of the University Hospital of Ulm 1–6 days after parturition. Mothers were being screened for childhood maltreatment (CM) using the Childhood Trauma Questionnaire (CTQ). All participating mother–child-dyads are followed up twice: 3 months (t1) and 12 months (t2) after birth. Main outcomes are the infants’ psychological (disorganized behavior), physiological (autonomous nervous system [ANS] and hypothalamic–pituitary–adrenal [HPA] axis) stress reactivity.

### The sample

In total, 1460 women were approached for study participation in the maternity unit of the Ulm University Hospital. Exclusion criteria were age < 18 years, prematurity (under 37 weeks of pregnancy), insufficient knowledge of the German language, severe complications during parturition or health problems of mother and/or infant, current drug consumption or a history of psychotic disorders or current infections. 533 signed an agreement for participation and completed the screening interview (t0). 240 mother–child-dyads could be recruited for a follow-up 3 months (t1) after birth in laboratory as well as in home visit. For the second follow up (t2) 12 months after birth 247 mother–child-dyads followed the invitation and participated in a further laboratory and home visit. The reasons for the drop-out of mother–child-dyads from the beginning of the study to the last measurement point (t2) varied and ranged from personal reasons, lack of interest to missing time windows for carrying out investigations. In order to enable the largest possible sample even at the last measurement time (t2), mother–child-dyads were also included at the last measurement time (t2), even though an investigation of the dyads at t1 was not possible.

For 163 mother–child-dyads we could complete the ANS data measurement all over the SSP thus these 163 mother–child-dyads were included in the following analyses. Missing data sets were due to non-divorcing spot electrodes in 23 cases of mother (n = 4) and child (n = 19). In addition, 14 infants refused to place on the wireless lightweight mobile units with seven disposable spot electrodes on their skin and 9 measurements could not be analyzed because of motion artifacts. For our analysis, we only considered complete data sets of mother–child-dyads.

Mothers’ age at time of measurement was in between 19 and 43 years (mean 32.5 years [SD 4.4 years]). The body mass index (BMI) of the investigated mothers were between 17.3 and 48.9 (mean 24.4 [SD 5.1]). 78.5% of the mothers were married or living in a partnership. 89.6% of the mothers had German citizenship. The level of education within the sample was comparable to the educational background of the German population (2014 Federal Statistical Office 2015). 0.6 % had no school diploma, 28.2% a basic secondary school degree (9 years of school), 12.9% a secondary school degree (10 years of school) and 57.7% a grammar school degree (13 years of school). Furthermore, 28.2% of the mothers had medical risk factors e.g. chronic disease, high blood pressure or allergies. 83 male and 79 female infants were investigated in laboratory visit. All mother–child-dyads were examined around 12 months of infant’s age (12.0 ± 0.1 months) (Table 1).

**Table 1:** Descriptive analyses of covariates

		Disrupted	Non-disrupted	Chi^2^-test		
	N	Male (%)	Female (%)	Chi^2^	df	p
Infant sex	163	65.5	72.2	.84	1	.36

						Independent t-test		
	N	M	SD	M	SD	t	df	p
Mother’s age at laboratory visit	157	33.22	5.13	33.54	3.69	.43	155	.67

*Chi^2^-test

**Independent t-test

All personal data (like perceived stress of the mothers, BMI, level of education or medical risk factors) as well as the perceived stress questionnaires were analyzed by paper-and-pencil questionnaires. The mothers were asked to complete them before and after the SSP. Some mothers were asked to answer the questionnaire at home and send them back by mail because of growing impatiens of the babies. Five mothers did not complete and send back the questionnaire.

### Procedures

12 months (t2) after birth all mother infant-dyads were invited for a laboratory visit in order to investigate mothers’ and infants’ stress reactivity in relation to their quality of interactive behavior. Therefore, mother and infant were invited from 10.00 a.m. to 1.00 p.m. to the Department of Child and Adolescent Psychiatry/Psychotherapy, University Hospital of Ulm. After a resting phase of approximately 15–20 min including a short small talk between mother and the test administrator about the procedure of investigation, mother and infant were asked to place on wireless lightweight mobile units (Mindware Technologies, Gahanna, USA) with seven disposable spot electrodes on their skin. Before starting the Strange Situation Procedure (SSP) mother and infant listened to a digitally recorded lullaby to calm down (episode 1). After the SSP all mothers were asked to fill in questionnaires about parental stress (Parenting Stress Index) [63], psychological stress (Perceived Stress Scale) [64] as well as families support and service provision. The quality of maternal interactive behavior was videotaped during the SSP between mother and infant and was analyzed with the “Atypical Maternal Behavior Instrument for Assessment and Classification (AMBIANCE)” (AMBIANCE; [65]). Based on the theory of Main and Hesse [66], Lyons-Ruth and colleagues developed the “AMBIANCE, to assess anomalous parental behavior of mothers’ during interactions with their infant. In addition to the frightened, frightening, and dissociated parental behavior described by Main and Hesse [66, 67], Lyons-Ruth and colleagues also consider profound disruptions in mother-infant interaction as well as behaviors that are physically or emotionally withdrawn [68]. The AMBIANCE scale is coding disrupted maternal behaviors on five dimensions: *affective communication errors, role/boundary confusion, disorganized/ disoriented behaviors, negative/intrusive behavior, and withdrawal*. Behaviors on each of the dimensions are coded on a 7-point scale and an overall score of the level of disruption is determined. The level of disrupted communication was assigned based on the frequency and intensity of all disrupted behaviors mothers displayed in the course of the interaction with their infant. A level of disrupted communication up to 4 is considered “not-disrupted” and a level from 5 to 7 is considered “disrupted”. A single coder scored all play sessions and was blind to all other data of the mother–child-dyads. This coder was trained by and reliable with the original developers of the AMBIANCE [65].

### ANS measures and SSP

To measure ANS reactivity wireless lightweight mobile units (Mindware Technologies, Gahanna, USA) were used to record ECG and ICG simultaneously and continuously in the infant during the SSP. HR, RSA and LVET were determined as follows: HR was derived from the measurement of the interbeat-intervals using the ECG. RSA is determined from the interbeat-intervals of the ECG and the respiratory rates derived from the ICG. LVET results from the time interval during systole until the closure of the aortic valve, derived from the ICG. HR, RSA und LVET were determined continuously while realizing the standardized protocol of 7 episodes in SSP (e2–e8). Additionally we added a 3 min time interval before starting the SSP to get a baseline measure from mother and infant. Therefore mother and infant listened to a digitally recorded lullaby (Brahms’ Lullaby) while infant was sitting on mothers lap (e1) SSP episodes in detail were: (e1) baseline to normalize the neuroendocrine and catecholaminergic stress axes in infant and mother, (e2) mother and infant were alone in the room with the infant exploring the room and the mother sitting on a chair, (e3) first encounter and interaction with the stranger, (e4) mother went out of the room (first separation), (e5) mother came back after a time period ranging from 30 s and 3 min dependent on child’s irritation and reaction of being separated from the mother (stranger left the room while reunion), (6) mother left the room for the second time while infant is alone in the room (second separation), (e7) the stranger came back instead of the mother, (e8) the mother came into the room (second reunion) while the stranger went out of the room [57].

Before analyzing the ANS data we filtered and scored them using the mindware software (BioLab 3.1 1.0J; Mindware Technologies, Gahanna, USA). Artifacts derived from child’s movements, speech or close physical contacts were eliminated. Every segment of the data was checked and corrected for inaccurate R-peak detections by trained coders [57]. Each of the 8 episodes were divided into segments of 30 s. Finally the first six segments of 30 s of each episode were used for statistical evaluation. If there were less than 6 segments available all present data was used. The data cleaning procedures, including surveillance at random were adapted to previously described procedures.

### Statistical analyses

We conducted statistical analyses using Statistical Package for the Social Sciences version 23.0 (SPSS Inc., Chicago, IL). Statistical significance was set at p < .05. For multiple testing of Pearson correlations the Bonferroni correction has been applied. Normal distribution of data was tested by non-parametric Kolmogorov–Smirnov test. Since data were normally distributed, analyses were analyzed as follows: ANOVA for repeated measures was calculated for each physiological data variable (HR, RSA, LVET) between subjects (group: “not-disrupted”/“disrupted” maternal behavior, mother, infant) and within subjects (for episode 1 to 8). Greenhouse–Geisser correction for repeated measures was applied. Infant sex, age of the mother at birth as well as perceived stress of the mother were entered as covariates. Episode × group interactions was calculated between the current and the preceding episode (e.g. e1 to e2).

## Results

### Descriptive analyses

Descriptive statistics are shown in Table 1. No significant differences were detected between the “non-disrupted” and the “disrupted” maternal behavior group concerning infant sex, mother’s age at laboratory visit and perceived stress and were therefore not considered for further analyses.

In the AMBIANCE overall score of ‘non-disruptive’ vs. ‘disruptive’ maternal behavior 68.7% of the investigated mothers showed ‘non-disruptive’ behavior. 31.3% of the mothers showed ‘disruptive’ behavior in interaction with their infant on a level from 5 to 7 (see Table 2).

**Table 2:** Allocation of the AMBIANCE overall score and subscales in ‘non-disruptive’ vs. ‘disruptive’ maternal behavior

	Frequency	Valid percent	Cumulative percent
AMBIANCE overall score			
‘Non-disruptive’	112	68.7	68.7
‘Disruptive’	51	31.3	100.0
Total	163	100.0	
AMBIANCE subscale: affective communication errors			
‘Non-disruptive’	135	82.8	82.8
‘Disruptive’	28	17.2	100.0
Total	163	100.0	
AMBIANCE subscale: role/boundary confusion			
‘Non-disruptive’	159	97.5	97.5
‘Disruptive’	4	2.5	100.0
Total	163	100.0	
AMBIANCE subscale: disorganised/disoriented behaviors			
‘Non-disruptive’	149	91.4	91.4
‘Disruptive’	14	8.6	100.0
Total	163	100.0	
AMBIANCE subscale: negative/intrusive behavior			
‘Non-disruptive’	156	95.7	95.7
‘Disruptive’	7	4.3	100.0
Total	163	100.0	
AMBIANCE subscale: withdrawal			
‘Non-disruptive’	141	86.5	86.5
‘Disruptive’	22	13.5	100.0
Total	163	100.0	

### Maternal behavior and child’s ANS

**Fig. 1:**
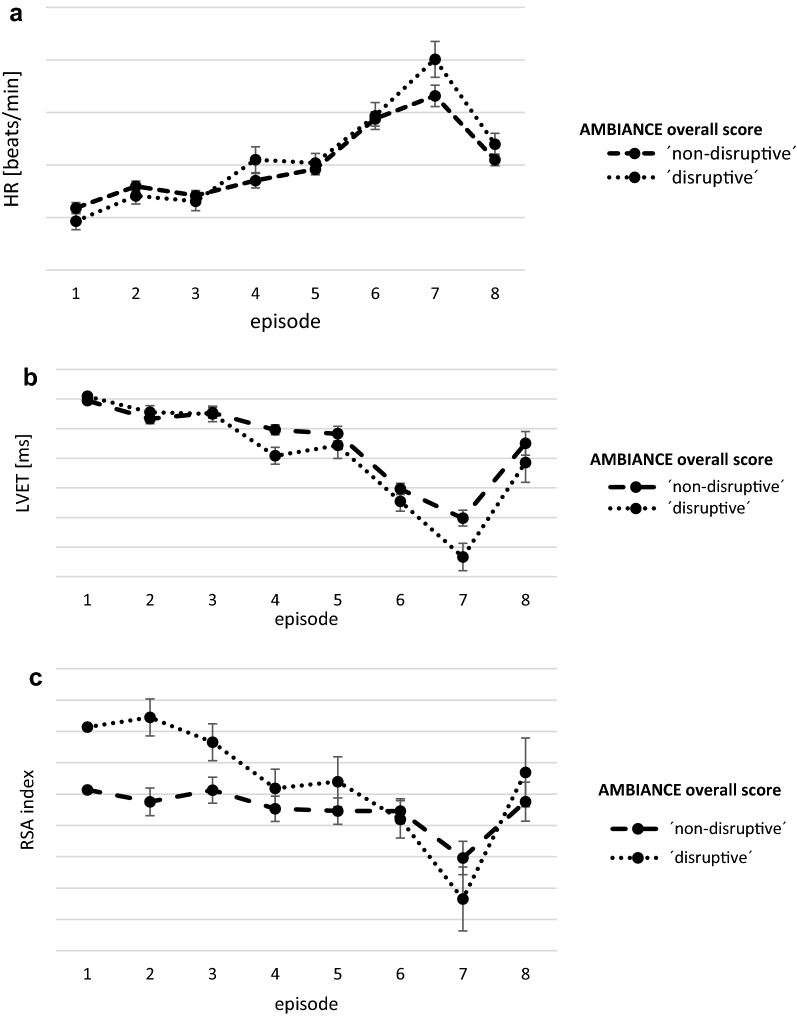
**a** ANOVA for repeated measures for the AMBIANCE overall score of ‘non-disruptive’ vs. ‘disruptive’ behavior and child’s HR. **b** ANOVA for repeated measures for the AMBIANCE overall score of ‘non-disruptive’ vs. ‘disruptive’ behavior and child’s LVET. **c** ANOVA for repeated measures for the AMBIANCE overall score of ‘non-disruptive’ vs. ‘disruptive’ behavior and child’s RSA

Values for HR, RSA and LVET of the infant analyzed with the ANOVA for repeated measures depending on the classification of maternal disruptive or non-disruptive behavior are shown in Table 3 and Fig. 1a–c.

**Table 3:** ANCOVA for repeated measures for AMBIANCE overall score of ‘non-disruptive’ vs. ‘disruptive’ behavior and child’s ANS (HR, RSA and LVET)

Source	Type III sum of squares	df	Mean square	F	Sig.
HR_time-effects					
Greenhouse–Geisser	72,281.10	2.82	25,653.7	109.6	.00
HR_group-by-time effects					
Greenhouse–Geisser	2532.74	2.82	898.91	3.84	.01
Erorr (HR)					
Greenhouse–Geisser	106,212.66	453.63	234.14		
HR_main group effects	57.68	1	57.68	.46	.50
RSA_time-effects					
Greenhouse–Geisser	62.17	4.12	15.08	15.18	.00
RSA_group-by-time effects					
Greenhouse–Geisser	15.65	4.12	3.80	3.82	.00
Error (RSA)					
Greenhouse–Geisser	659.25	663.64	.99		
RSA_main group effects	1.13	1	1.13	2.12	.15
LVET_time-effects					
Greenhouse–Geisser	240,395.00	2.95	81,529.21	84.93	.00
LVET_group-by-time effects					
Greenhouse–Geisser	6754.33	2.95	2290.71	2.39	.07
Error (LVET)					
Greenhouse–Geisser	455,699.98	474.72	959.93		
LVET_main group effects	604.78	1	604.78	1.73	.19

### ANOVA for repeated measures: HR depending on maternal behavior

Significant group-by-time effects on HR focusing on the overall score of the AMBIANCE were detectable ((F (2.82, 453.63) = 3.84, p = .01)). For main group effects (F (1, 161) = 0.46, p = .50) no differences could be shown (see Table 3; Fig. 1a).

### ANOVA for repeated measures: RSA depending on maternal behavior

For infants’ RSA group-by-time effects (F (4.12, 663.64) = 3.82, p = .00) were highly significant. For main group effects no differences could be shown (F (1, 161) = 2.12, p = .15) (see Table 3; Fig. 1b).

HR and RSA of children of mothers are significantly altered depending on the maternal interacting behavior.

### ANOVA for repeated measures: LVET depending on maternal behavior

LVET values showed marginal but not significant differences neither for group-by-time effects nor for main group effects [(group-by-time effects (F (2.95, 474.72) = 2.39, p = .07) main group effects (F (1, 161) = 1.73, p = .19) (Fig. 1c)].

LVET values of the child showed marginal differences depending on the quality of maternal interacting behavior.

As it could be shown that the two groups of children differ in relation to the HR, RSA and LVET by trend, the next step is to investigate in which specific episodes exactly the two groups of children differ. For more detailed analyses the differences between episodes were examined using mixed ANOVA for repeated measures. Especially infants of mothers with disruptive interacting behavior showed a significant increase in HR when the stranger came in (e3) and the mother went out of the room (e4) (F (1, 161) = .01, p = .01) and from e6 to e7, when mother left the room and the stranger came back, (F (1, 161) = 4.15, p = .04) compared to infants of mothers without disruptive behavior. For RSA we could show significant differences between e2 to e3 when mother and infant were alone up to the first encounter with the stranger, (F (1, 161) = 5.11, p = .03)) and e7 to e8, when the stranger came back instead of the mother and the second reunion with the mother (F (1, 161) = 3.76, p = .05) (Fig. 1b). Infant’s LVET of mothers with disruptive behavior showed a significant decrease in LVET when the stranger came in (e3) and the mother went out of the room (e4) (F (1, 161) = 4.08, p = .05) in contrast to infants of mothers with ‘non-disruptive’ behavior.

### Correlation analyses

Pearson correlation analyses between the AMBIANCE overall score and child’s HR, RSA and LVET revealed significant results. Children’s HR was correlated with the AMBIANCE overall score in e2 (r(163) = .21, p = .01), e4 (r(163) = .18, p = .02) and e7 (r(163) = .19, p = .02) indicating that HR increases in relation to a better maternal behavior mainly in episodes in contact with the stranger. Child’s RSA showed a positive relation to the score of maternal behavior in e2 (r(163) = .21, p = 0.01) indicating that vagal response increases with higher score of maternal behavior when mother is near to the child (e2).Child’s LVET was negatively correlated with the AMBIANCE scores in e4 (r(163) = − .18, p = .02), e7 (r(163) = − .19, p = .01) and e8 (r(163) = − .17, p = .03) indicating that LVET shortened (= increase in SNS activity) with increasing score of maternal behavior in contact with the stranger (e4, e7) and during reunion with the mother (e8).

## Discussion

Our findings revealed specific ANS changes in 12-month-old infants in relation to the quality of maternal interacting behavior. Infants of mothers with disruptive behavior showed increased HR when leaving alone with the stranger with a consecutive increase in SNS, reflected by a decrease in LVET. PNS decreased in contact with the stranger and increased during mother and child reunion. HR increased with increasing AMBIANCE scores when the infant was left alone with the stranger, induced by LVET which was negatively correlated with the AMBIANCE scores. These results indicate that disruptive maternal behavior results in an increase in child’s SNS activation, especially in contact with the stranger. To sum up, disruptive behavior of the mother leads to an autonomic imbalance with SNS predominance in the 1-year-old child.

### Infant’s PNS regulation in relation to maternal disruptive behavior

It could be shown that an impaired maternal interacting behavior quality effects the offspring’s stress reactivity reflected by significant PNS changes. It is known that PNS measured by RSA is a key indicator of regulation [51]. Infants of disruptive mothers showed higher variations in RSA values over all episodes. This is in good accordance with the results of Gunnar et al. [42] who detected that lower quality of maternal behavior was related to a higher activation in RSA. In our study, from playing with the mother up to stranger’s encounter (e2 to e3), infants of disruptive mothers showed a decrease, while infants of mothers without disruptive behavior showed an increase in RSA. It seems that the infants with disruptive mothers experienced a PNS discharge at the time being alone with the mother until the first contact with the stranger, while infants with non-disruptive mothers showed PNS activation. This is in line with Moore et al. [9] who detected disruptive behavior as a predictor for decreases in RSA and argued that environmental demands of infants could be buffered by sensitive caregiving leading to an increase in RSA [9, 43–46]. Mothers with disruptive behavior may be less able to buffer the onset of stress for their child by a stranger than mothers without disruptive behavior, which is reflected by a decrease in child’s RSA. From the entering of the stranger while mother is expected (e6–e7) up to second reunion with the mother (e7 to e8), those infants with disruptive mothers showed a maximum in RSA alteration that may be interpreted as an autonomic sign of great relief due to reunion with the mother compared to the infants with non-disruptive mothers. In contrast to that, using the Still-Face Paradigm (SFP; [41]) Enlow et al. [47] reported that lower maternal sensitivity during periods of stress were associated with lower PNS and higher infant’s SNS activation at 6 months of age [9, 47]. However, infants in our sample were older (range 10–15 months) and it is well known that the mode of autonomic imbalance in response to stressful situations depends on the age of the child [35]. Therefore, results are difficult to compare. Our findings indicate that being alone with a stranger while mother is expected may trigger stress especially for those infants with a lower secure base of maternal sensitivity.

### Infant’s SNS regulation in relation to maternal disruptive behavior

Especially in stressful situations like the ‘first encounter with the stranger’ (e3) up to mothers leaving (e4), infants of mothers’ with disruptive interacting behavior showed a significant increase in HR. The increase in HR was induced mainly by the SNS branch of the ANS, reflected via the LVET, which showed a significant decrease in infants with disruptive mothers compared to infants with non-disruptive mothers. One may state that disruptive mothers were less supportive or sufficient for reassurance and less “regulative” than sensitive mothers. This is in line with Thompson and Trevathan [69], who found that infants’ HR was reduced depending on the responsiveness and sensitivity of caregiving. They demonstrated that infants of mothers with a higher quality of caregiving could better regulate their own stress compared to infants of mothers’ with a lower level of caregiving [69].

The preejection period (PEP) has been widely used to measure SNS in attachment research and emotional as well as cognitive related measurements [54, 55]. Only few studies are available that investigated SNS in infants in relation the quality of maternal caregiving. Roder et al. (in press) have shown that PEP was not suitable in distinguishing SNS changes during SSP in the 1-year-old child. However, LVET has been identified as appropriate measure to detect SNS changes, since LVET is a frequency-related measure and therefore suitable for young children (Roder et al. in press). The lack of PEP related differences was confirmed by Enlow et al. [47] who showed that maternal interacting behavior was not associated with any SNS differences in infants in the 1st year of life. PEP in young infants revealed significant stability over time [56] that lead to the hypothesis that alterations in PEP might be discovered later in childhood [56]. This is in good accordance with findings of differences in child’s SNS response measured by PEP from the age of 3 years [54]. Therefore, in our study, we used LVET as an alternative, frequency-related SNS measure instead of PEP. LVET has been shown to decrease in response to stress [38, 39, 70]. However, there is only little research on LVET measures in young infants [53]. Most studies on child’s LVET are realized using echocardiography. In these studies, systolic time intervals and HR are closely correlated, however, less important in infants than in adults [71]. The measurement of LVET allows to reflect child’s chronotropic SNS reactivity which might be a more sensitive SNS marker for stress in young infants compared to PEP [57]. To our knowledge LVET in infants depending on maternal interacting behavior has not yet been investigated.

In summary, focusing on the maternal quality of interacting behavior, our analyses showed that the most important episodes in SSP of particular ANS importance were those episodes with contact to a strange person. It became evident that it was not decisive for ANS response whether the infant was separated from the mother or not. Rather, the appearance of a stranger seemed to trigger ANS stress reactions in which maternal interaction behavior appeared to be a relevant predictor buffering stressful situations and reducing the child’s emotional arousal. Inadequate or anomalous maternal behavior affects child’s PNS and SNS stress responses early in life resulting in an increase in HR in stressful situations like an unexpected encounter of or being in contact with a strange person. Our findings highlight the regulatory function of the maternal interacting quality for child’s physiological regulation in stressful situations in the early years of life. Regarding the role of maternal caregiving with respect to the child’s ANS extends our understanding of the impact that parenting may have. Inadequate or anomalous maternal behavior could inhibit the development of infants’ regulatory strategies, which could be a risk for later stress-related mental and physical burden and may be linked to increased stress vulnerability and difficulties in emotion regulation [72, 73].

## Limitation

Limitations of this study were missing data sets due to non-divorcing spot electrodes in 23 cases of mother (n = 4) and child (n = 19). This explains the differences in sample size of mothers and infants. In addition, it would be important to include a second coder for scoring maternal behavior focusing further analysis. Accordingly, the analyses of maternal behavior could be supported by an international reliability. Apart from that, the demographic characteristics (like a comparatively high level of maternal education) and geographic location of the sample limits the study’s generalizability. This has to be taken into account when comparing our results with other studies with populations with different demographic characteristics.

## Conclusion

Caregiving quality in early life may influence the responsiveness of the SNS and PNS branches of the ANS. Our findings suggest that maternal disruptive interacting behavior may have an effect on in child’s physiological regulation, particularly in response to stressful challenges of social interaction, i.e. being with a strange person. The findings highlight the importance of supporting high quality caregiving as a resilience factor for child’s development of vagal balance. Warm and sensitive maternal interacting behavior enables to buffer stressful situations and may be considered as nurturing and protective which is likely to promote later psychophysiological health.

## Data Availability

The datasets analysed during the current study are available on a database of the University Hospital of (Ulm).

## Abbreviations

ANS: autonomic nervous system
PNS: parasympathetic nervous system
SNS: sympathetic nervous system
HR: heart rate
RSA: respiratory sinus arrhythmia
LVET: left ventricular ejection time
PEP: pre-ejection period
ECG: electrocardiograms
ICG: impedance cardiograms
AMBIANCE: Atypical Maternal Behavior Instrument for Assessment and Classification
SSP: strange situation procedure
